# Petrified child mummies by Paolo Gorini (19th century CE, Lodi, Lombardy, Italy): anthropological, pathological, and conservation perspectives

**DOI:** 10.3389/fmed.2025.1692282

**Published:** 2025-12-18

**Authors:** Omar Larentis, Ilaria Gorini, Michele Campus, Stefano Vanin, Enrica Tonina, Giuseppina Carta, Alberto Carli, Lisa De Luca, Dario Piombino-Mascali

**Affiliations:** 1 Bagolini Laboratory of Archaeology, Archaeometry, Photography (LaBAAF), Department of Humanities, University of Trento, Trento, Italy; 2 Centre of Research in Osteoarchaeology and Paleopathology (CROP), Biotechnologies and Life Sciences Department (DBSV), University of Insubria, Varese, Italy; 3 Superintendence for Archaeology, Fine Arts, and Landscape for the Provinces of Como, Lecco, Monza-Brianza, Pavia, Sondrio and Varese, Milan, Italy; 4 Fujifilm Healthcare Italia, Cernusco sul Naviglio, Milan, Italy; 5 Earth, Environment and Life Sciences Department (DISTAV), University of Genoa, Genoa, Italy; 6 Department of Humanities, Social Sciences and Education (SUSeF), University of Molise, Campobasso, Italy; 7 Cranfield Forensic Institute, Cranfield University, Cranfield, United Kingdom; 8 Department of Anatomy, Histology and Anthropology, Faculty of Medicine, Vilnius University, Vilnius, Lithuania

**Keywords:** mummies, non-adults, preservation, anatomical collection, X-ray

## Abstract

This study presents an interdisciplinary analysis of six non-adult petrified specimens prepared by the Italian scientist Paolo Gorini (1813–1881) in Lodi, Lombardy, during the 19th century. Housed since 1981 in the Old Hospital, these individuals represent the entire known corpus of Gorini’s preserved children. The research combined macroscopic inspection, radiographic imaging, anthropological assessment, and entomological observations to document biological characteristics, embalming techniques, and conservation needs. Radiographic analysis enabled the estimation of ages at death, ranging from approximately 1.5–12 months, and provided detailed information on skeletal development, dental formation, and pathological conditions. Soft tissues were preserved to an exceptional degree, allowing for the identification of dermal, muscular, and visceral structures. Notable modifications, such as intraorbital inserts, revealed Gorini’s attention to appearance and presentation. The results demonstrate the effectiveness of Gorini’s petrification process, now better understood through recent discoveries of his embalming formulae. His technique achieved both anatomical preservation and long-term stability, even in fragile non-adult individuals. Beyond technical achievements, the specimens reflect broader 19th-century cultural attitudes toward childhood, mortality, and commemoration in a period of elevated death rates. By integrating biological, historical, and conservation perspectives, this study contributes both to the documentation of a unique anatomical collection and to the safeguarding of its future. It also situates Gorini’s work within the scientific and cultural milieu of his time, highlighting the intersection of experimental anatomy, public display, and the desire for permanence over death.

## 1 Introduction

Research on non-adult mummies is a subject that combines archaeology, anthropology, and the study of ancient cultural practices (1). While mummification is most often associated with adults, the preservation of children and infants reveals important insights into the beliefs, values, and rituals of past societies. Examining these practices allows researchers to better understand not only technical aspects of body preparation but also broader social and emotional dimensions related to childhood, mortality, and the afterlife (2–5).

Mummification, whether deliberate or accidental, is a phenomenon found across different cultures and geographical regions [e.g., (6–11)]. In the case of non-adults, intentional embalming raises questions about the significance attributed to children within ancient communities. In some societies, children were considered spiritual beings with unique roles in religious thought, while in others they were seen as incomplete members of society whose deaths carried different symbolic meanings (12). The decision to embalm a child, therefore, reflects both the technological capacity of a community and its underlying worldview regarding life, death, and continuity beyond the grave (13).

From a technical perspective, the embalming of non-adults often reveals a surprising level of care and sophistication. Techniques varied depending on time and place, but they could include evisceration, desiccation, wrapping, and the application of resins or other preservatives (2, 11, 14, 15). In some instances, the small size of the body may have made preservation more difficult, requiring adjustments in technique compared to those used for adults (16). Scientific analyses, including radiography, computed tomography, and chemical testing of tissues, have provided new information about how these processes were carried out and what substances were used (3, 11, 17, 18). Such methods also allow scholars to distinguish between intentional embalming and natural mummification caused by environmental conditions such as extreme dryness or cold.

Beyond the technical dimension, non-adult mummies raise broader anthropological and ethical considerations [e.g., (19, 20)]. They highlight the vulnerability of children in past societies, where high mortality rates made infant death a common experience. At the same time, the decision to preserve these small bodies indicates that their loss was not treated lightly (11). In many cases, the presence of grave goods, careful wrapping, and ritual markings suggests that non-adult mummies were objects of deep emotional and cultural investment [e.g., (21)]. Studying these remains today requires sensitivity, as they are not only scientific specimens but also once-living individuals whose preservation reflects grief, remembrance, and belief.

Research into non-adult preservation also contributes to our understanding of health and disease in antiquity. Many child mummies exhibit signs of malnutrition, infections, or congenital conditions, which provide valuable information about the challenges of childhood in different historical periods (4, 22–29). The ability to safeguard and study these remains thus extends far beyond ritual analysis, offering glimpses into patterns of health, family life, and medical knowledge through history.

In sum, the study of non-adult mummies is a multidisciplinary topic that bridges science, history, and culture. It raises fundamental questions about how human societies have dealt with mortality and the passage from life to death. By examining the practices and meanings associated with these remains, researchers can gain a deeper appreciation of the complexity of ancient attitudes toward children, death, and the possibility of existence beyond the physical body.

This study focuses on the children prepared by Paolo Gorini, analyzing their preservation and the cultural, scientific, and historical insights they offer.

## 2 Paolo Gorini and his collection

**Figure 1:**
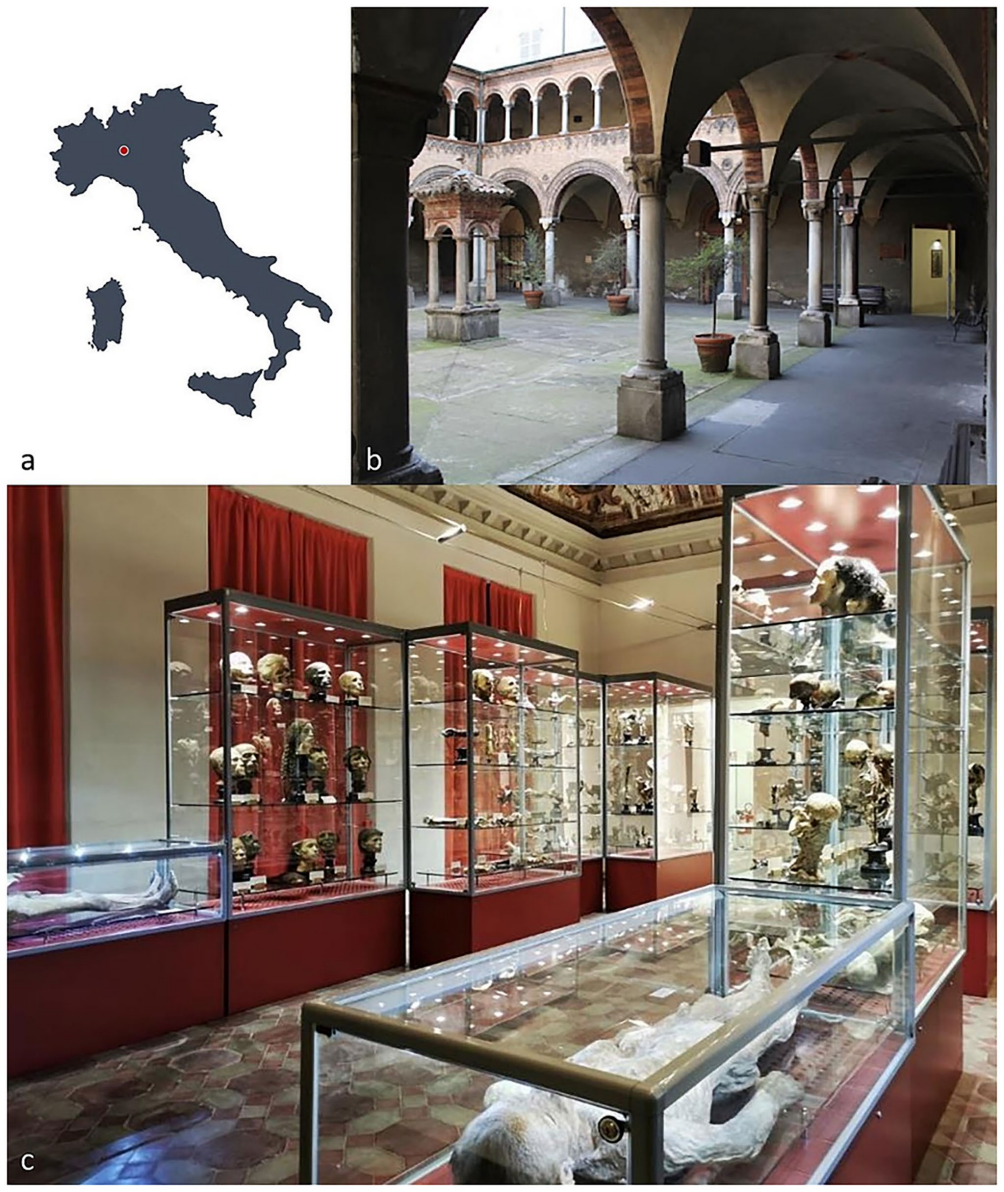
**(a)** Location of the Paolo Gorini anatomical collection in Lodi, Lombardy, Italy (45°18′49.1″N, 9°30′30.06″E). **(b)** The collection is housed in rooms adjacent to the cloister of the Old Hospital in Lodi, to which the heirs of the embalmer donated the specimens. **(c)** Today, the collection contains numerous petrified preparations displayed in showcases, including heads and anatomical sections, two whole-body specimens, and several dry preparations.

In the 19th century, the boundary between science, medicine, and public spectacle was often porous. Among the figures who exemplify this intersection is Paolo Gorini (1813–1881), an Italian physician, chemist, and natural philosopher whose reputation was built on his remarkable ability to preserve human remains through processes of petrification (29, 30). Gorini’s so-called “petrified specimens” occupy a unique position within the history of anatomical preservation (Figure 1). Unlike traditional embalming, which sought to prevent decomposition through chemical treatments or desiccation, Gorini’s method aimed to transform organic tissue into hardened, stone-like material, thereby ensuring both permanence and resistance to decay (30). His work reflects the scientific curiosity, cultural anxieties, and aesthetic sensibilities of the period, while also provoking questions that remain relevant in contemporary discussions of death, memory, and the treatment of the human body (31).

The process of petrification as practiced by Gorini, once a closely guarded secret, has since been revealed in its main components. Archival analyses show that he employed a precise mixture of mineral salts and chemical reagents that penetrated the organic matter of the corpse, producing a specimen that retained its external form while acquiring a stony consistency. Historical sources—including firsthand documents by Gorini’s associate Luigi Rovida—report the use of various chemical compounds, such as mercuric chloride, arsenous acid, sulfuric acid, spirits of wine, and slaked lime, mixed in variable proportions and applied differently depending on the formula and technique used (32–34). Instrumental analyses of several samples represent an initial systematic study of both the substances employed by Gorini and the products generated by their chemical reactions (33). Although the absence of published diffraction spectra limits more detailed evaluation, the macroscopic “chalky” consistency of the preparations appears corroborated by the presence of bi-hydrated calcium in the three analyzed samples.

In this sense, Gorini’s petrified bodies occupy a middle ground between fossilization, chemical embalming, and the artificial fabrication of anatomical models. They were simultaneously scientific objects, artistic creations, and, in some cases, public curiosities (31). The secrecy surrounding his techniques contributed to his mystique, earning him both admiration and suspicion among contemporaries.

Gorini’s work must also be understood within the cultural and intellectual climate of 19th-century Europe. Advances in anatomy and pathology had created an unprecedented demand for reliable means of preserving bodies for research and teaching (35, 36). At the same time, Romanticism and the rise of secular thought were reshaping attitudes toward death, leading to new forms of commemoration and memorialization (37). Gorini’s petrification can be interpreted as material responses to these needs: they provided durable specimens for scientific inquiry while also offering an alternative to traditional funerary practices. Indeed, Gorini himself famously embalmed the remains of Giuseppe Mazzini, one of the leaders of the Italian Risorgimento, thereby linking his scientific methods with national identity and political symbolism (38).

The case of the petrified specimens also illuminates broader tensions between science and spectacle. Gorini’s creations were exhibited in scientific settings but also drew popular attention as curiosities, sometimes provoking unease or outright controversy (30, 31). Their ambiguous status—oscillating between anatomical preparation and artistic artifact—raises important questions about the boundaries of medical science and the cultural uses of the human body. Even today, preserved specimens challenge modern audiences by blurring the line between education and morbidity, reverence and transgression (39).

Modern scholarship on Gorini’s petrified specimens is necessarily interdisciplinary, drawing on history of science, anthropology, art history, and cultural studies. While some details of his method remain uncertain, the significance of his work lies not only in the chemical transformation of tissue but also in the symbolic transformation of the human body into an enduring object. These specimens embody 19th-century aspirations to master nature, confront mortality, and preserve memory through technology. They also foreshadow later debates surrounding body preservation, from plastination in the late 20th century to contemporary bio-art practices (40, 41).

In this light, the petrified specimens of Paolo Gorini should not be regarded merely as historical curiosities. They are instead complex cultural artifacts that reveal how science, technology, and society intersect in the ongoing human effort to negotiate the inevitability of death. By studying them, scholars gain insight not only into 19th-century scientific experimentation but also into the broader cultural imagination that sought permanence in the face of impermanence.

This study is part of the project “Under the Skin: New Secrets of the Gorini Collection of Lodi,” which investigates a selection of previously unpublished anatomical preparations by Paolo Gorini. These specimens, never before analyzed and currently preserved in the anatomical collection of the former Hospital of Lodi, represent an unexamined source of information on Gorini’s preservation techniques. In addition to advancing historical and scientific understanding, the project also contributes to the planning of future conservation strategies, some of which have already been applied to portions of the collection, particularly the embalmed busts.

For the analyses, data were collected from both untreated specimens and those that had previously undergone conservation work. This comparative approach allowed for an initial assessment of the impact of conservation by contrasting the original condition of the specimens with their current state. Conventional digitized radiography was employed to obtain biological information on the petrified bodies, focusing on skeletal age and pathological conditions, while also evaluating the effectiveness, mechanisms, and outcomes of Gorini’s preservation methods. The study further tested the potential of next-generation portable radiography on petrified human remains, enabling comparisons with existing knowledge of Gorini’s techniques and revealing new details of the preparation process.

Radiographic investigations were complemented by entomological analysis, undertaken to expand knowledge about the conservation status of the specimens and to identify potential evidence of decomposition processes prior to petrification. Insects play a fundamental role in the decomposition of human bodies. Species of the order Diptera (flies) are typically the first to colonize a corpse shortly after death, whereas species of Coleoptera (beetles) and Lepidoptera (clothes moths) are more commonly associated with later stages of colonization. The dynamics of insect activity depend on both intrinsic and extrinsic variables, including the stage of decomposition, accessibility of the body, and the presence of repellent substances. The presence of insects on mummified remains is also well documented, as their feeding activities leave distinct traces: maggots, beetles, and clothes moths often produce holes in soft tissues, while ants (Formicidae) can leave serpiginous surface scratches. The detection or absence of such traces provides valuable information on peri- and post-mortem events, which can be applied in both forensic and archaeological contexts (42–44). Carta et al. (45) provide a notable archaeological example of using maggot-induced holes to assess funerary practices. Studying 17th–19th century mummified remains of three hermits, they demonstrated the authenticity of the clothing by analyzing the co-occurrence of holes on both skin and garments and inferred the probable season of death based on maggot species. In a forensic context, Byard and Heath (46) reported a case in which ant abrasions revealed the cadaver’s body position. Research has also focused on beetles—particularly larder and carpet beetles (Coleoptera: Dermestidae) [e.g., (47, 48)]—though, as summarized by Viero et al. (44), a wide range of insects can alter the body, clothing, and surrounding scene.

## 3 Materials and methods

**Figure 2:**
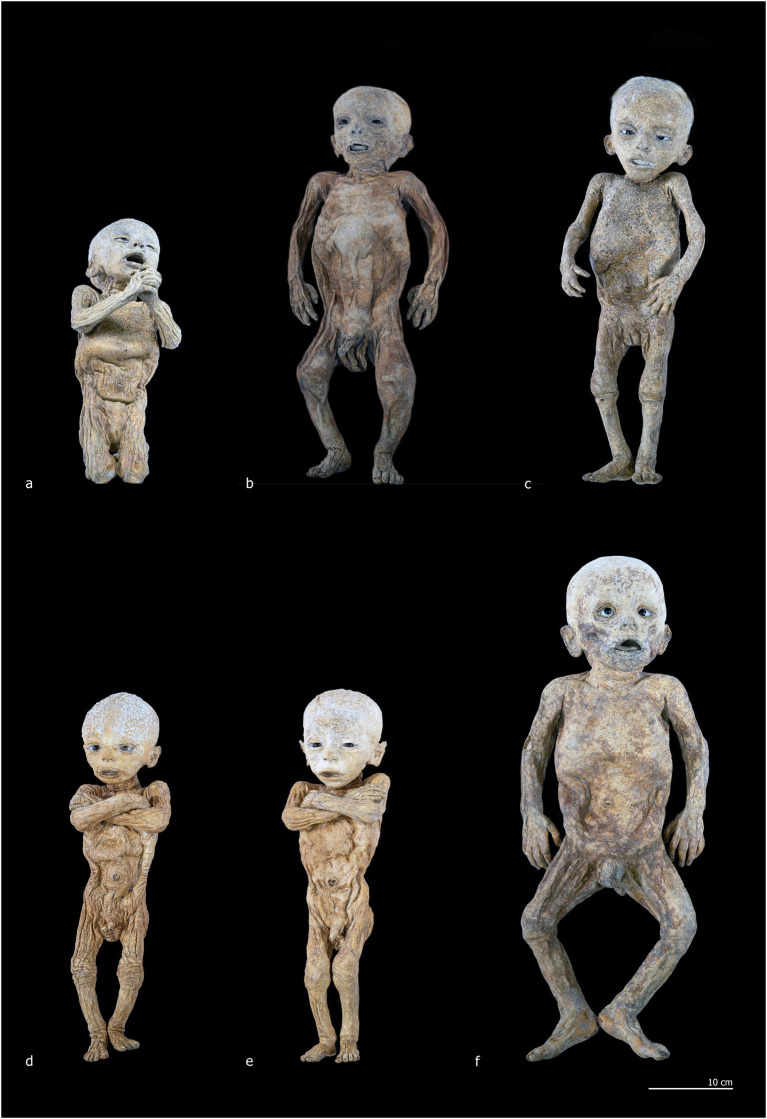
Corpus of non-adult subjects petrified by Paolo Gorini during the 19th century. **(a)** Individual 1 (ID1); **(b)** Individual 2 (ID2); **(c)** Individual 3 (ID3); **(d)** Individual 4 (ID4); **(e)** Individual 5 (ID5); **(f)** Individual 6 (ID6).

The sample includes the entire known corpus of non-adult individuals petrified by Paolo Gorini during the 19th century (Figure 2). It consists of six non-adult subjects that have been housed since 1981 in the anatomical collection of the Old Hospital in Lodi. These bodies were recently subjected to conservation-oriented restoration, aimed at cleaning and stabilizing the superficial patinas accumulated over time and consolidating dermal tissue where necessary, in order to safeguard the overall integrity of the specimens. The six individuals were macroscopically examined and systematically documented through both general overview and detailed photography. Primary sexually dimorphic characteristics were recorded to determine biological sex. The analysis also included a photo-documented assessment of taphonomic and diagenetic features, the embalming techniques employed, and the positioning of the bodies.

A mobile digital radiographic system (Fuji model FDR Xair) was installed in the exhibition rooms of the Gorini Collection, where the mummies are seen. The embalmed bodies, currently displayed in large glass cases, were carefully removed and transferred to an adjacent room for radiographic examination. This procedure minimized handling, reduced the risk of transportation-related damage, and limited exposure to changes in temperature and humidity. Radiographs were acquired using the FDR Xair detector, the FDR D-Evo III, which is equipped with a CsI scintillator and Fujifilm technologies such as a uniquely positioned TFT layer and a plastic rather than glass substrate. These features increase sensitivity to low radiation doses and provide enhanced spatial and contrast resolution, supported by advanced image post-processing. Standardized projections and exposure parameters were employed as follows: skull/cervical spine, 62 kV—1.6 mAs; thoracic/dorsal spine, 64 kV—2 mAs; pelvis/lumbar spine, 64 kV—2 mAs; lower limbs, 60 kV—1.6 mAs; and upper limbs/shoulder, 58 kV—1.6 mAs. The resulting images were evaluated with FLOSS DICOM software—specifically, Weasis Medical Viewer version 4.5.1—by an interdisciplinary team including radiographers, biological anthropologists, paleopathologists, and historians of medicine specializing in Gorini’s preservation methods.

Following the radiographic investigations, anthropological examinations were conducted to estimate age at death, assess bio-anthropological features, and identify pathological conditions, interpreted using both clinical and paleopathological literature (49–52). Age estimation was based on measurements of long bones, the presence and development of secondary ossification centers, and the eruption and development of teeth (53–56). The latter parameter was prioritized, as dental elements are less affected by growth-related stressors that can delay skeletal development and potentially lead to underestimation of chronological age. In addition, an entomological survey was performed, alongside an evaluation of tissue degradation, and a preliminary assessment of conservation needs for the specimens.

All anthropological investigations were carried out in accordance with the guidelines of the Italian Central Institute for Archaeology (*Istituto Centrale per l’Archeologia*, ICA) and the Central Institute for Cataloguing and Documentation (*Istituto Centrale per il Catalogo e la Documentazione*, ICCD) (57). The research was formally authorized by the Italian Ministry of Culture (*Ministero della Cultura*, MIC), through the Superintendence for Archaeology, Fine Arts and Landscape (*Soprintendenza Archeologia, Belle Arti e Paesaggio*, SABAP) for the provinces of Cremona, Mantua, and Lodi.

## 4 Results

### 4.1 Morphology

**Figure 3:**
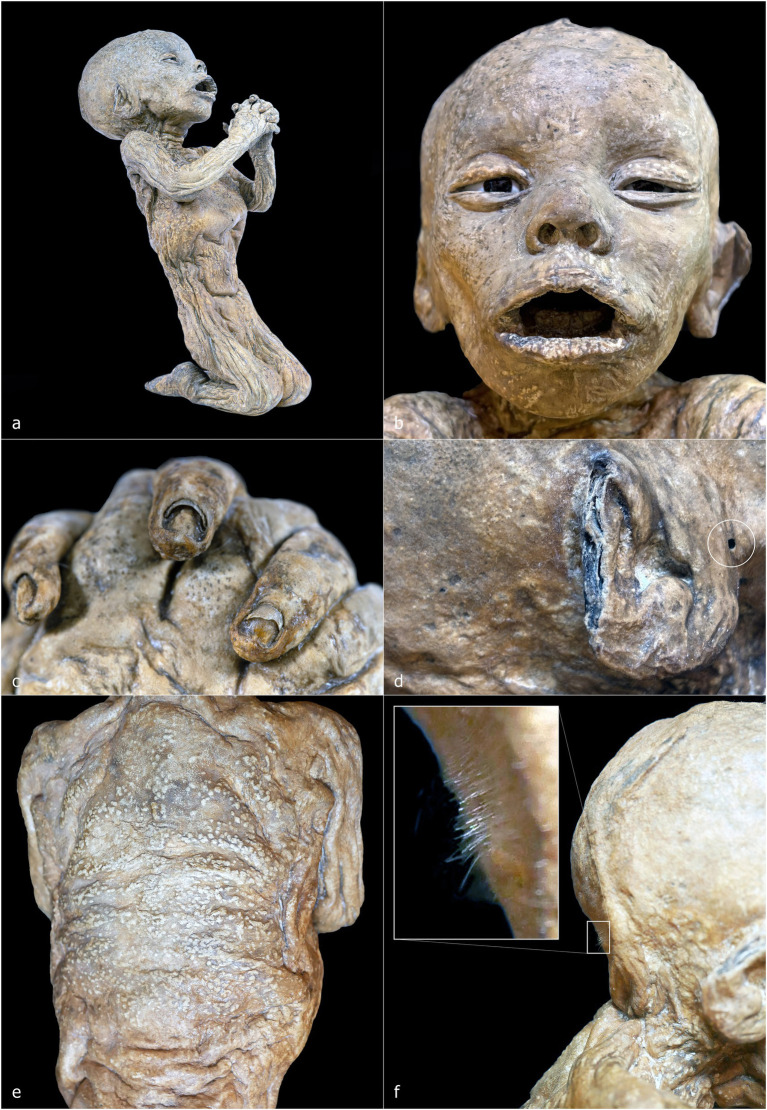
ID 1—detailed photographs: right lateral view **(a)**; frontal view of the face **(b)**; close-up of the hands **(c)**; close-up of the right ear **(d)**; posterior view of the back **(e)**; right lateral view of the nape with detail of the hair system **(f)**.

ID1 is a non-adult female (♀) individual. The mummified body is preserved in a kneeling position, measuring 32 cm from the knees to the top of the head and approximately 42 cm in total length from feet to head (Figures 2a, 3a). The subject is positioned with knees close together and feet in hyperextension, allowing full contact of the dorsal surfaces of the legs with the supporting plane. The back forms a particularly straight axis and is inclined backward, creating an angle of approximately 60° between the torso and feet. The upper limbs are flexed and raised from the torso, with the hands together in a prayer posture in front of the chin. The head is slightly tilted posteriorly, the mouth open, the nose flattened relative to its natural anatomy, and the eyelids arranged to reveal partially open eyes (Figure 3b).

The ventral cavity is flattened (Figure 3a), whereas the dorsal skin rests on a virtual plane (Figure 3e). In the nuchal region, the skin accumulates over the occipital area (Figure 3f). The dermis exhibits the characteristic ocher-brown pigmentation of Gorini’s preparation, although the coloration is heterogeneous, with darker and lighter patches; the lighter areas appear as small, rounded elevations beneath the skin surface, predominantly on the back. Facial skin appears comparatively smooth and uniform, contrasting with the more irregular and folded skin of other regions.

The auricular pavilion is partially absent, with frayed, irregular margins (Figure 3d). Fingernails and nail beds are exceptionally well preserved, as is the hair, which shows variable lengths in several scalp regions (Figures 3c,f). A circular perforation of 2.2 mm in diameter is present near the right ear (Figure 3d, white circle).

**Figure 4:**
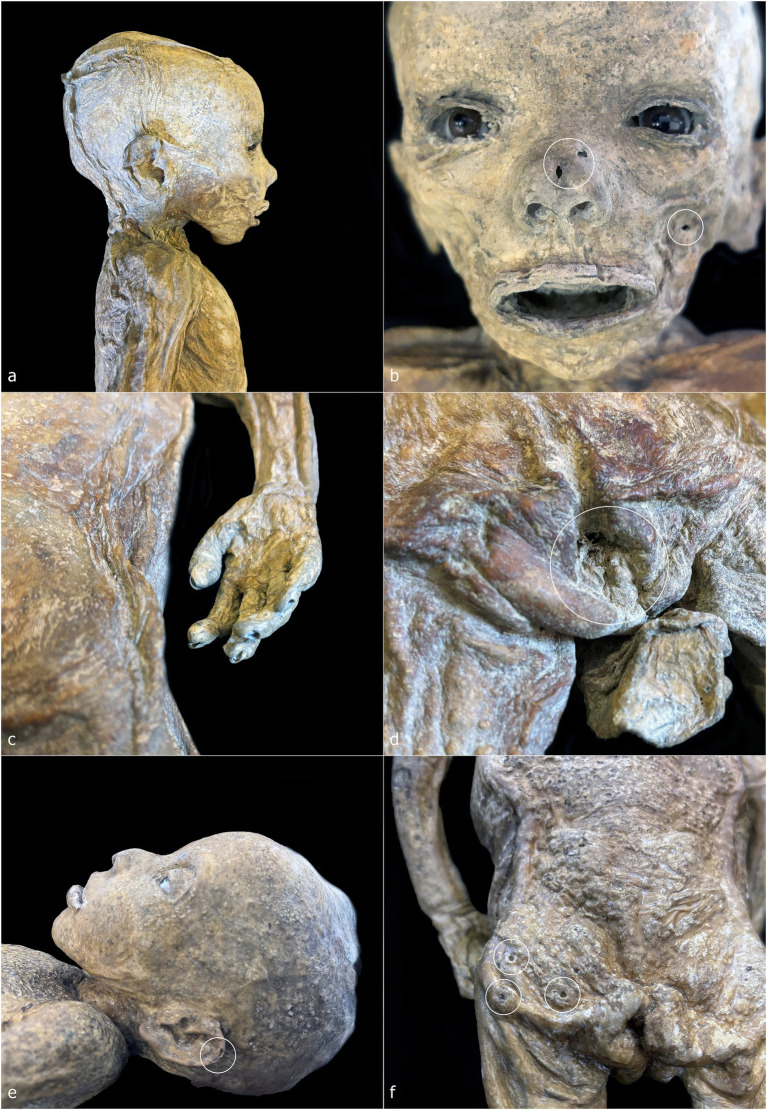
ID2—detailed photographs: right lateral view of the head and chest **(a)**; frontal view of the face **(b)**; detail of the skin on the right flank and right hand **(c)**; detail of the perineal area **(d)**. ID3—detailed photographs: left lateral view of the head **(e)**; posterior view of the gluteal region **(f)**.

ID2 is a non-adult male (♂) with a total length of 48 cm, embalmed in a supine position (Figures 2b, 4a). The body is horizontally aligned, with the dorsal surfaces of the feet forming a 90° angle with the legs, which are slightly abducted. The shoulders are retracted posteriorly, and the arms lie parallel to the chest. The eyes and mouth are slightly open, and the nose is flattened (Figure 4b). The abdomen is flattened, and the dermis exhibits heterogeneous coloration: ocher-brown areas interspersed with lighter patches, particularly on the torso, upper limbs, and head. The skull exhibits a sub-rectangular outline with prominent frontal bosses. Facial skin is smoother than the body, though the mouth and eyelid margins lack natural anatomical definition. Nail beds, fingernails, and hair are preserved, with hair closely cropped but showing roots and numerous shafts. Multiple circular or ellipsoidal holes (2.1–3.4 mm diameter) are present across the face and, to a lesser extent, the body (Figure 4b, white circles). The anal opening and surrounding sphincter tissues are visible and frayed (Figure 4d, white circle).

ID3 is a non-adult female (♀), embalmed supine, with a total length of 49 cm (Figures 2c, 4d). The body lies horizontally, with feet at 90° relative to the legs; the right leg shows outward rotation. Shoulders are retracted, and the forearms rest above the abdomen. The head tilts slightly toward the left shoulder, with eyes and mouth open, and a flattened nose. A white, chalky substance is visible at the left corner of the mouth. Facial skin is tight, contrasting with wrinkled skin at the knees, armpits, and posterior torso and gluteal regions. Hair is well preserved, up to 3.2 cm on the scalp (Figure 4e). Tissue loss affects the left auricle (Figure 4e, white circle). Circular depressions with raised rims are present on the posterior body, forming donut-like lesions (Figure 4f, white circles). Fine granular wrinkling occurs on the head and upper back, with typical ocher-brown Gorini pigmentation elsewhere (Figures 4e,f).

**Figure 5:**
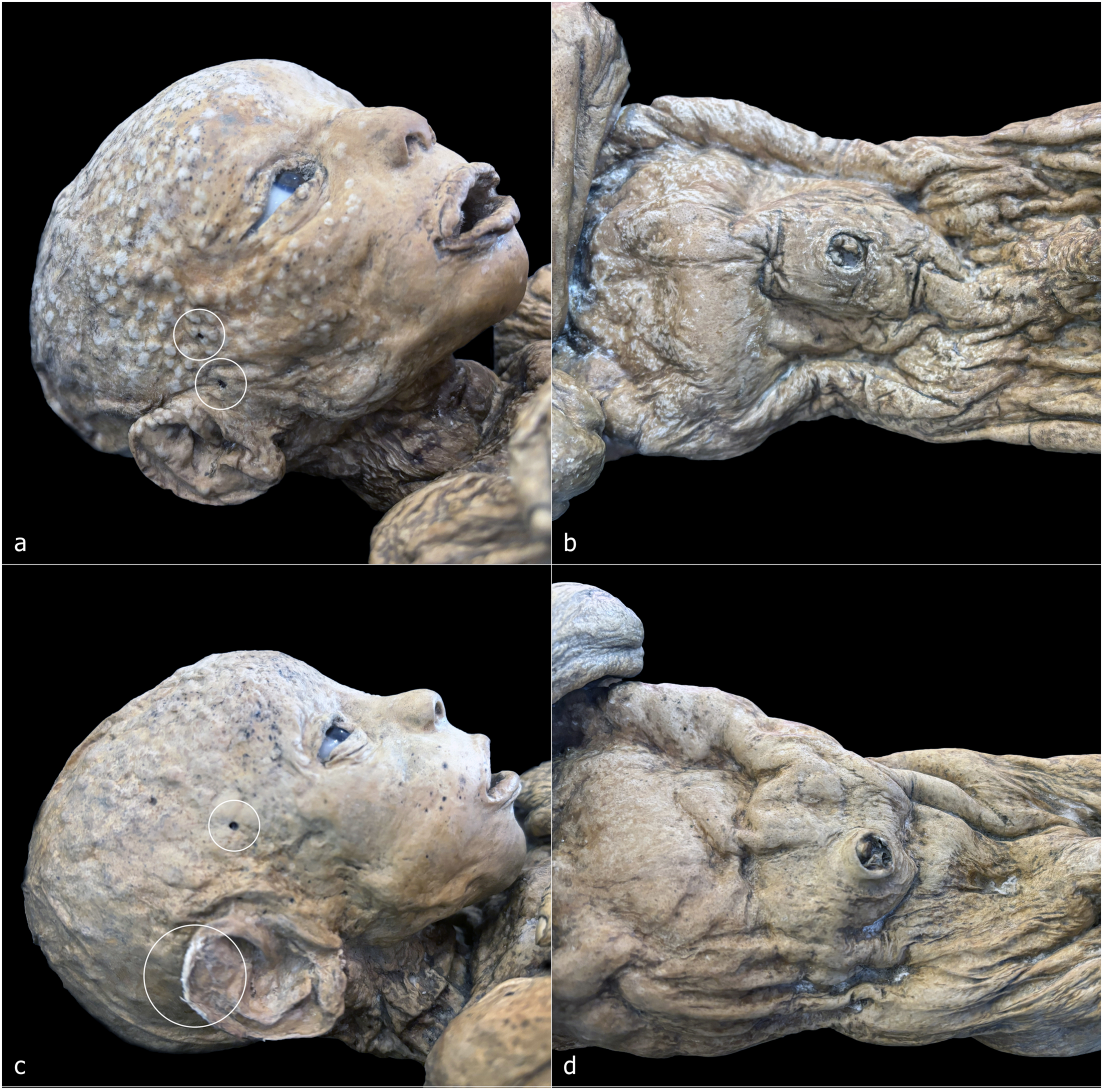
ID4—detailed photographs: right lateral view of the head **(a)**; anterior view of the torso **(b)**. ID5—detailed photographs: right lateral view of the head **(c)**; anterior view of the torso **(d)**.

ID4 and ID5 are non-adult males (♂) showing strong similarities in size, embalming position, petrification outcomes, and preservation (Figures 2d,e). Both bodies were supine: ID4 measures 44 cm and ID5 45 cm from feet to head. Legs are generally parallel, with ID4 showing slight separation and ID5 a mild lateral rotation of the right leg. Arms are crossed over the chest: in ID4, the right arm lies over the left, with hands positioned beneath or on the opposite arm; in ID5, the configuration is reversed. Heads align with the sagittal plane, with slight anterior tilt. Both abdomens are sunken, and the skin shows extensive wrinkling, though the facial regions are smoother (Figures 5a–d). Eyes and mouth are open in both individuals. ID4 exhibits flattened nasal structure; ID5 retains more anatomical nasal preservation. Auricular defects are present in ID5 (Figure 5c, lower white circle). Circular perforations (2.0–2.9 mm) are scattered across the face and body (Figures 5a,c, white circles). Skin color ranges from light brown to ocher, with ID4 displaying additional discoloration and raised areas on the cranium. Umbilici are well preserved in both individuals (Figures 5b,d).

**Figure 6:**
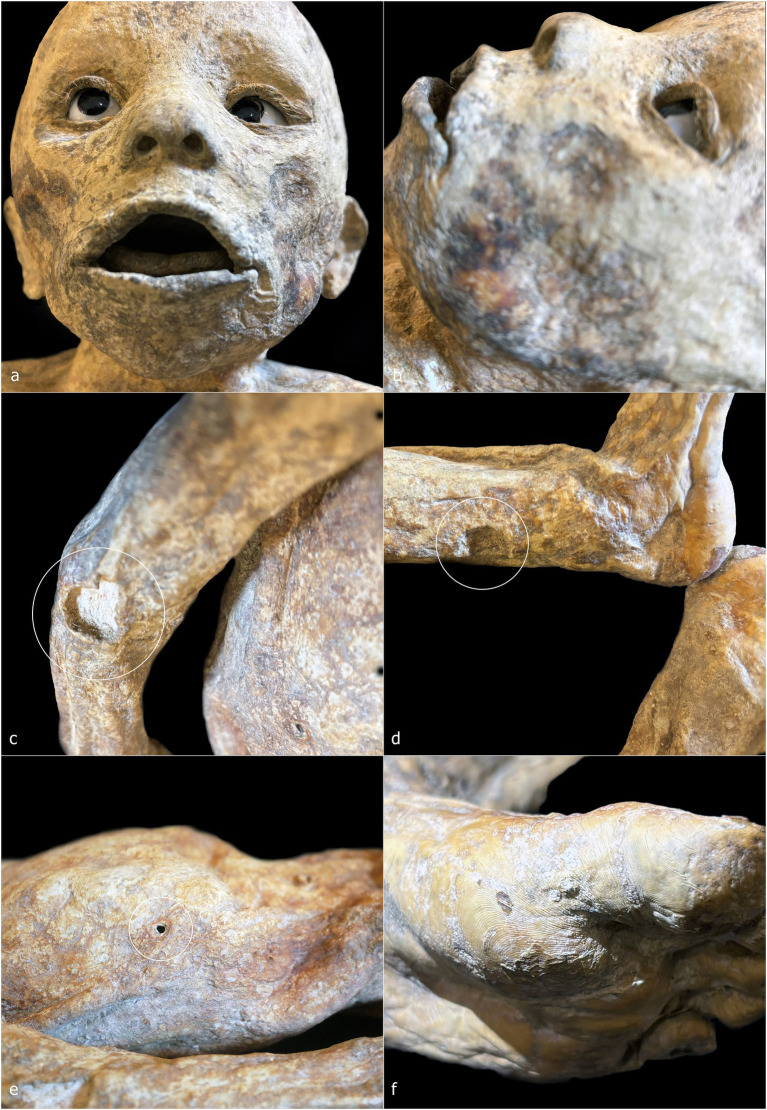
ID6—detailed photographs: frontal view of the face **(a)**; left lateral view of the face **(b)**; detail of the right arm and forearm **(c)**; detail of the left leg and ankle **(d)**; right lateral view of the trunk **(e)**; plantar surface of the left foot **(f)**.

ID6 is a non-adult male (♂), supine, with a head-to-foot length of 61 cm (Figure 2f). Legs and ankles are laterally rotated. The shoulders are slightly retracted; with arms parallel to the torso. The eyes and mouth are slightly open; the nose is flattened. The abdomen is flattened, and the dermis shows heterogeneous coloration and texture. High-detail areas include eyelashes and plantar dermatoglyphics (Figures 6a,f). Other regions are less preserved. Small circular lesions with raised or irregular margins are present on the right arm (2.9 × 2.1 mm), above the left ankle (2.5 × 1.8 mm), and on the right side of the torso (6.0 × 5.1 mm) (Figures 6c–e, white circles).

### 4.2 Radiology

**Figure 7:**
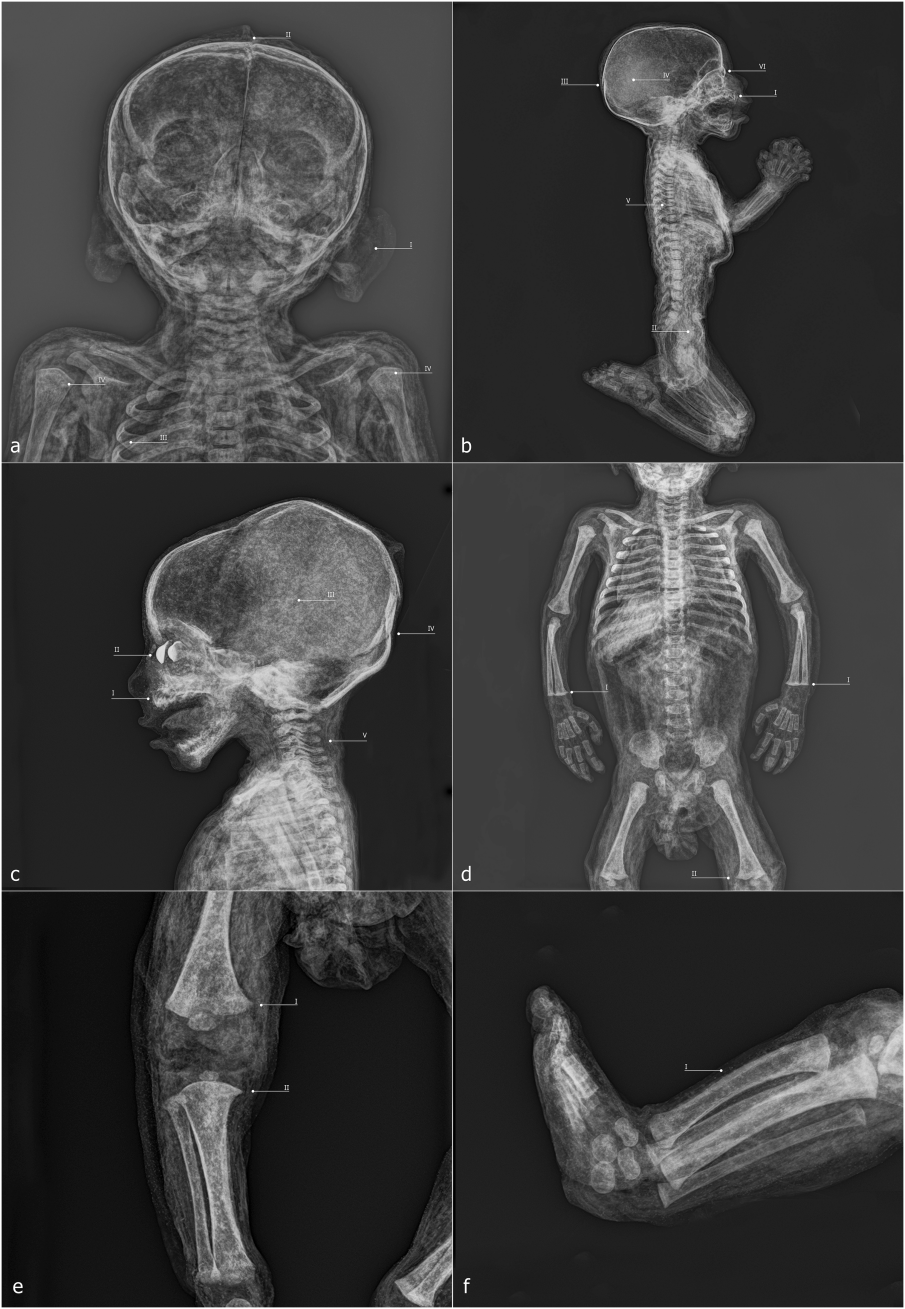
ID1—radiographic images: anteroposterior view of the head and shoulders **(a)**; full-body laterolateral view **(b)**. ID2—radiographic images: laterolateral view of the skull and shoulders **(c)**; anteroposterior view of the trunk and upper limbs **(D)**; anteroposterior view of the right leg **(e)**; laterolateral view of the legs and feet **(f)**.

ID1 is an individual with a skeletal age estimated between 2 and 5 months postnatal, based on skeletal development and dental formation, which are clearly visible in the lateral X-ray projection. The high quality of the imaging allows, for example, verification of the presence and developmental stage of the upper incisors, as well as the thin bony layer closing the dental crypt (Figure 7bI). The soft tissues are well preserved and clearly readable, including cartilaginous structures such as the auricles, dermis, muscle structures, and internal organs (Figure 7aI). Greater radiodensity is observed in the thoracic and visceral regions, where circumscribed areas of particularly high density can be identified (Figure 7bII). Within the skull, there is a slight disconnection between the occipital bone and the adjacent bones (Figures 7aII,bIII), and the posterior portion of the skull appears more radiodense than the anterior portion (Figure 7bIV). The skin of the neck, showing evident folds in its posterior area, envelops a spine that is in perfect anatomical connection. In the mid-thoracic region, the vertebral bodies exhibit bone sequester on both their anterior and posterior portions (Figure 7bV). The sternal ends of the ribs appear slightly expanded (Figure 7aIII), and a line of higher radiodensity than the surrounding bone crosses both humeri parallel to the metaphyseal plate, approximately 1.7 mm from it (Figure 7aIII). The only foreign elements visible within the body are the eyes, which appear as two convex medallions with higher radiodensity compared to the surrounding soft tissues, particularly in the iris area (Figure 7bVI).

ID2 is an individual approximately 6–9 months postnatal. Here, too, the evaluation of maximum diaphyseal length, the stage of appearance and fusion of ossification centers, and dental development can be assessed using X-ray imaging (Figure 7cI). At the cranial level, two particularly radiodense areas are visible within the orbital cavities (Figure 7cII), along with increased radiodensity in the posterior neurocranium (Figure 7cIII) and discontinuities in the superior portion of the occipital squama (Figure 7cIV). The spine is in anatomical connection along its entire length (Figure 7cV). In the forearms, the distal surfaces of the radius and ulna are flattened (Figure 7dI), a feature also observed in the distal femora (Figures 7dII,eI,eII). Finally, the lateral projection of the legs shows a slight curvature of the tibial diaphysis (Figure 7fI).

**Figure 8:**
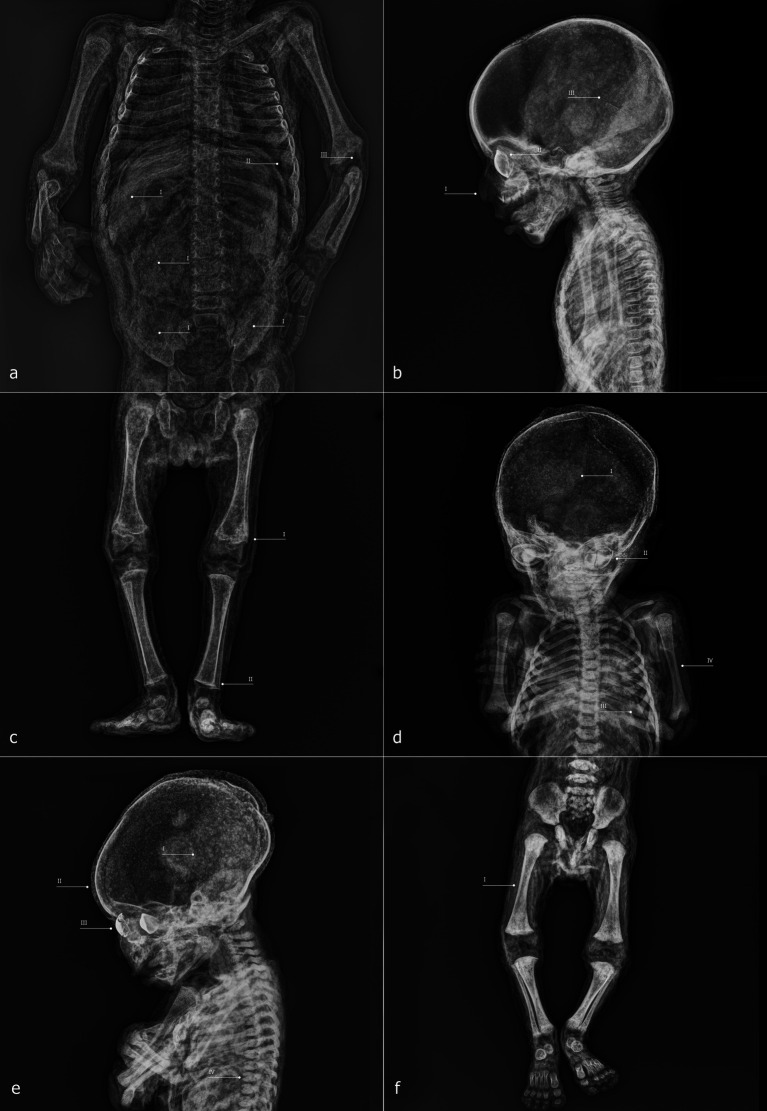
ID3—radiographic images: anteroposterior view of the trunk and upper limbs **(a)**; laterolateral view of the skull and torso **(b)**; anteroposterior view of the lower limbs **(c)**. ID4—radiographic images: anteroposterior view of the skull and trunk **(d)**; laterolateral view of the skull and torso **(e)**; anteroposterior view of the lower limbs **(f)**.

ID3 has a skeletal age estimated at approximately 3 months postnatal. In this case as well, the acquisitions are highly readable, with clear recognition of soft tissues, including the viscera, where certain elements appear highly radiodense (Figure 8aI), and the face, where nasal soft tissues are appreciable (Figure 8bI). As observed in the other specimens, two radiodense ovoid elements are visible within the orbital cavities (Figure 8bII), and increased radiodensity is present in the nuchal region of the neurocranium (Figure 8bIII). The sternal ends of the ribs appear slightly expanded (Figure 7aII), while the distal articulations of the radii and femora near the metaphysis show irregular margins and non-homogeneous density (Figures 8aIII,cI). The distal epiphyses of the tibiae and fibulae have slightly expanded margins, which cause them to appear flattened (Figure 8cII).

ID4 is an individual with an estimated age of approximately 1.5 months postnatal. Within the neurocranium, a more radiodense area is visible in the posterior region on lateral projection (Figures 8dI,eI), and several small radiodense areas, already noted during macroscopic analysis, are present within the dermal layer (Figure 8eII). The elements inside the orbital cavities are clearly visible; the left one shows three discontinuities (Figures 8dII,eIII). The rib extremities appear slightly expanded (Figure 8dIII), and the long bones show good mineralization (Figures 8dIV,fI). In the mid-thoracic spine, some horizontal fissures of the vertebral bodies are observed (Figure 8eIV).

**Figure 9:**
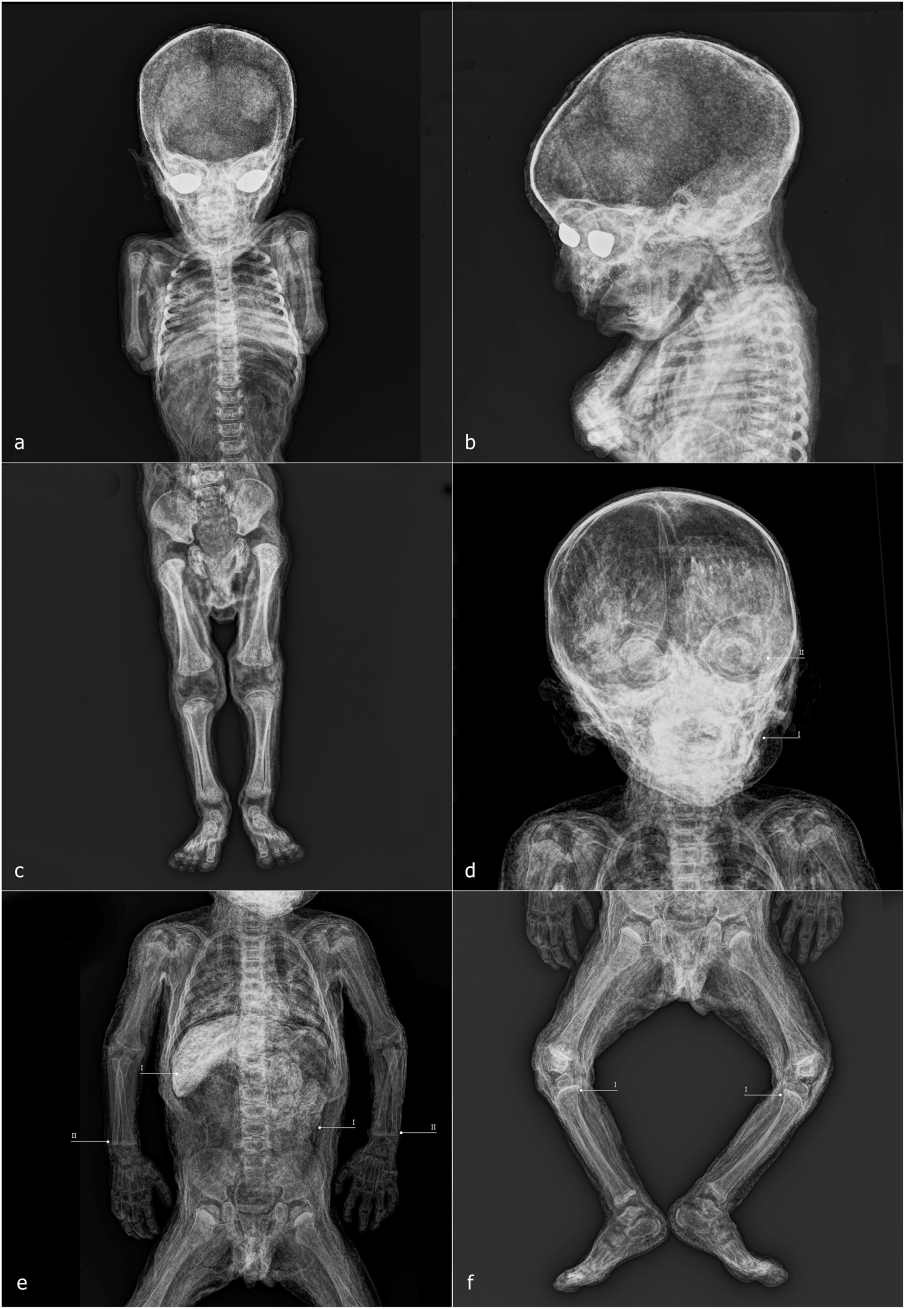
ID5—radiographic images: anteroposterior view of the skull and trunk **(a)**; laterolateral view of the skull and shoulders **(b)**; anteroposterior view of the lower limbs **(c)**. ID6—radiographic images: anteroposterior view of the skull and shoulders **(d)**; anteroposterior view of the trunk and upper limbs **(e)**; anteroposterior view of the lower limbs **(f)**.

ID5 is also a subject of approximately 1.5 months, and presents a condition entirely similar to that of ID4, sharing all the aforementioned characteristics except for the orbital cavity, where the intraorbital discs remain intact (Figures 9a–c).

ID6 is an individual estimated to be approximately 9–12 months postnatal, mummified in a supine position. The specimen exhibits high radiodensity, particularly in the facial region (Figure 9dI) and in the abdominal area, where distinct structures with recognizable morphology can be observed within the abdominal cavity (Figure 9eI). The increased radiodensity of the face makes it more challenging to identify the ovoid elements within the orbital cavities, compared to other specimens (Figure 9dII). The distal epiphyses of both radii and ulnae display a radiodense line parallel to the metaphyseal plate, located approximately 2.7 mm from it (Figure 9eII). Similar lines are also present approximately 4.3 mm from the proximal metaphyseal plate of both tibiae (Figure 9fI).

### 4.3 Entomology

All of the holes and skin alterations described in the previous paragraphs were inspected. Their shape, depth, and the characteristics of the hole ridges were evaluated. The average size of the holes ranges from 2 to 3.5 mm and, except for ID3, they are isolated and do not form clusters. In ID3, four main circular depressions are present on the right gluteus, with a fifth located on the posterior side of the leg.

The margins of the holes are smooth, and no signs of mandibular lesions are evident, such as those associated with maggot exit holes caused by skin enlargement. Indeed, these types of alterations typically consist of several clustered holes, often of uniform size, although occasionally varying depending on the larval stage. Carta et al. (45) provide a detailed description, including iconographic support, of maggot-induced holes in skin and textiles, which can be used for comparison with the holes observed on Gorini’s children. In contrast, the alteration of keratinous substrates by beetles and clothes moths is discussed and illustrated by Mazzarelli et al. (58). In both cases, the shape, size, and rim of the resulting damage differ markedly from the holes observed in the children analyzed in this study.

## 5 Discussion

Among the non-adult specimens petrified by Paolo Gorini during the 19th century are both male and female individuals, with estimated ages ranging from approximately 6 weeks to 9–12 months. Age estimation was conducted using three well-established anthropological methods: the measurement of diaphyseal length in long bones, the appearance and degree of development of secondary ossification centers and their fusion with primary centers, and—most importantly—the developmental stage and eruption pattern of the dentition.

Conventional digitized radiography, performed with state-of-the-art portable equipment, enabled the evaluation of these features even though Gorini’s preparation method replaced soft tissues with highly radiodense compounds which could potentially compromise the readability of certain diagnostic traits (59, 60). Sex does not appear to have been a selection criterion. However, younger age may have been a preferred variable for Gorini. This choice could have been driven by multiple factors.

Firstly, small body size likely yielded more successful results with Gorini’s petrification technique, making the treatment of non-adult bodies easier than that of adults. Indeed, these smaller bodies plausibly played a crucial role in refining his method, as adult specimens were likely produced later. Secondly, it is documented that Gorini transported some of his petrified children across Europe to demonstrate the effectiveness and reliability of his technique (29, 61). Finally, we might speculate that high infant mortality in the first year of life during this period would have ensured a steady availability of cadavers for Gorini’s experiments. While in some cases the preparations may have reflected the wishes of grieving families—such as in the widespread Victorian practice of memorializing deceased infants—Gorini more frequently appears to have worked on unclaimed bodies or those originating from the rural Lodi area (62). An additional, though unverifiable, hypothesis is that Gorini may have used the bodies of unbaptized infants, which is plausible given that in 19th-century rural Italy baptism was not always administered immediately after birth (50). The presence of a specimen of approximately 1 year of age is therefore consistent with this interpretation.

Some considerations must be made regarding the health status of these individuals. In ID1 and ID6, clear evidence of Harris lines is visible, particularly on long bones such as the tibia and femur. These transverse sclerotic bands detectable via radiography indicate interruptions in bone mineralization during endochondral ossification, reflecting disruptions in normal growth processes (63). Although the precise etiology of Harris lines remains debated, several studies associate them with adverse conditions such as systemic inflammation, malnutrition, excessive alcohol exposure, and various pathological states (64). The most reliable methods for estimating the age at which stress episodes occurred are based on the tibiae and femora (65, 66). However, in ID1, a Harris line is visible in the proximal humeri, while in ID6, pronounced lines are present in the distal radii and ulnae. Given the uncertainty associated with applying these methods to bones other than the tibia and femur, we propose interpreting these lines simply as markers of physiological stress experienced during the subjects’ short lives (67).

Harris lines are not the only indicators potentially associated with health conditions. In ID1 and ID3, the sternal surfaces of the ribs appear expanded—a condition that, when particularly pronounced, is referred to as rachitic rosary (49, 50). In ID2, the metaphyseal growth surfaces of the distal radii and ulnae, the distal femora, and the proximal tibiae are flattened. Similarly, in ID3, flattening is evident in the distal tibiae and fibulae, while the distal metaphyseal surfaces of the humeri and femora are poorly defined, possibly indicating reduced radiodensity in actively forming bone. Additionally, the tibiae of ID2 exhibit mild diaphyseal bowing. Frontal bossing was also recorded. These characteristics fall within the range of non-specific signs suggestive of micronutrient deficiencies, particularly involving vitamins C or D (4, 49, 50, 68–70). Rickets and scurvy, caused by deficiencies of vitamin D and vitamin C, respectively (71, 72), have received considerable scholarly attention. Characteristic bone manifestations of both diseases can co-occur in the same individual, a phenomenon termed “co-occurrence disease,” defined by Brickley and Mays (73) as a situation “where multiple (pathological) conditions occur simultaneously” [see also (68, 69, 74, 75)]. Such co-occurrence is reported primarily in non-adults aged approximately 3 months to 5 years (49, 74, 76). However, this interpretation remains tentative in the absence of further data, such as analysis of external cortical bone or radiographically inaccessible areas. It is also important to emphasize that the etiology of these skeletal changes may be multifactorial, depending on environmental, medical, or genetic factors acting individually or in combination (74, 77, 78).

Considering the geographic and social background of these individuals and the widespread presence of skeletal stress markers, it is plausible to interpret these findings as reflections of the living conditions and health status of the broader population (79). Additionally, weaning timelines are relevant. In most cases in 19th-century Italy, weaning occurred around 6 months of age, but specific circumstances—such as the absence of the mother or unavailability of wet nurses—could lead to earlier weaning, sometimes as early as 4 months (80–82). This allows us to hypothesize that the health conditions of the youngest individuals (ID1, ID3, ID4, and ID5) might reflect the nutritional and physiological status of their mothers or caretakers, linking observed deficiencies in children to an adult, female segment of the population (68, 83, 84).

Concluding the pathological observations, the thoracic vertebrae of ID1 exhibit deep cavitations in the vertebral bodies, visible in lateral radiographs. Although radiography does not allow precise assessment of their extent, these lesions appear more severe than Hahn clefts, which are an anatomical variant of the vertebral nutrient canal (85). Such cavitations are frequently associated with hematogenous spread of infection within the vertebral column (51, 52, 86). In light of this possible etiological variable, other diagnostic hypotheses must nonetheless be considered, such as the persistence of synchondroses. The latter, however, can be ruled out since the evidence does not concern only the vertebral bodies of the upper cervical tract (for example, between the body and the odontoid process of the axis, C2), but also those of the thoracic tract. Pathologies of an inflammatory or neoplastic nature must also be taken into account (87, 88). In summary, biological data on sex, estimated age, and evidence of physiological stress or pathological alterations are presented in Table 1.

**Table 1:** Summary of the anthropological and paleopathological data of the subjects.

ID	Sex	Age	Harris lines	Pathological features
1	F	2–5 months	Humeri 1.7 mm	Flattened sternal ends of the ribs; vertebral body cavities
2	M	6–9 months	/	Frontal bossing; expanded distal epiphyses of the radius and ulna; expanded distal femoral epiphyses; expanded proximal tibial epiphyses; tibial bending
3	F	3 months	/	Flattened sternal ends of the ribs; expanded distal tibial epiphyses; unmineralized distal epiphyses of the humeri and femora
4	M	1.5 months	/	/
5	M	1.5 months	/	/
6	M	9–12 months	Radii 2.7 mm; Ulnae 2.7 mm; Tibiae 4.3 mm	/

Table 1 shows that pathological evidence or signs of physiological stress were identified in all individuals except ID4 and ID5. This raises the question of why two skeletally “healthy” individuals came into Gorini’s possession (89). The two subjects are of similar age and skeletal features, but notably, they were petrified with their arms crossed and arranged symmetrically.

Typically, Gorini’s outcomes are highly heterogeneous in skin coloration, texture, and facial expression. However, these two children display nearly identical features, strongly suggesting they were treated in close temporal proximity, possibly simultaneously. Their pose may have been intentionally chosen to emphasize a symbolic or biological connection, suggesting they may have been twins who died prematurely. Gorini’s access to their bodies may have been due to both scientific relevance and familial misfortune.

Gorini’s preparation technique and the aesthetic outcomes provide additional insights. All subjects exhibit a flattened back, likely from lying supine on a smooth rigid surface. Increased radiodensity in the occipital area and dermal folds running parallel to the spine suggest horizontal positioning. Except for ID6, evidence exists of a second rigid surface shaping posture, for example, “hammer foot” configurations in ID2–5. Limb positions indicate that supports may have been used but physical restraint devices are absent.

Facial features reveal deliberate shaping. The eyes and mouths were molded using flat tools, with glass eyes inserted prior to tissue dehydration and hardening. This sequence indicates careful attention to facial realism to enhance the perceived success of the specimen. Glass eyes, likely locally sourced, reflect influences from other embalmers (90).

Luigi Rovida’s written descriptions once again shed light on the technique (29, 32). For non-adults, fluid perfusion was performed rectally, without incisions in the lower abdomen. Radiography confirms the absence of femoral arterial cannulation, supporting Rovida’s account. Evidence from ID2’s perineal region suggests invasive rectal perfusion, while internal organs remain *in situ*.

ID6 presents a unique case, with dermal loss at the right torso, right elbow, and left ankle, and higher overall radiodensity. These likely result from partial failure of the preservation process, with Gorini performing additional direct infiltration to salvage the specimen.

No evidence of early insect activity is visible on the bodies, suggesting both effective preservation of soft tissues and the absence of entomofaunal colonization between death and Gorini’s petrification process. Several factors, likely acting in combination, may account for this. One possibility is that the individuals died during colder seasons, when insect activity is naturally reduced, though it seems improbable that all deaths occurred exclusively in winter. Another explanation is that Gorini gained rapid access to the corpses and initiated petrification without delay, a scenario consistent with his connections to the hospital and his established collection of dry pathological specimens. Alternatively, the bodies may have been transferred quickly to sites with limited insect exposure, such as Gorini’s laboratories. While these hypotheses remain partly speculative, taken together they offer a coherent framework for interpreting the observed evidence.

Lastly, a relative temporal sequence can be hypothesized: ID6 as earliest, followed by ID2 and ID3, and finally ID1, ID4, and ID5 representing the most mature phase of Gorini’s activity, though this remains preliminary.

## 6 Conclusion

The interdisciplinary investigation of Paolo Gorini’s non-adult petrified specimens has provided new insights into both the biological features of the individuals and the embalming methods employed during the 19th century. Radiographic and anthropological analyses allowed us to estimate ages at death, identify pathological conditions, and recognize taphonomic and diagenetic alterations, while entomological observations offered insights in the absence of traces that contribute to the reconstruction of peri- and post-mortem processes. Together, these results demonstrate that Gorini’s practice of petrification achieved a remarkable degree of preservation, maintaining not only skeletal integrity but also soft tissues and internal structures.

From a technical perspective, this research confirms the effectiveness of Gorini’s embalming formula, recently identified through chemical analyses, and highlights its ability to preserve even fragile non-adult remains. The findings also illustrate Gorini’s meticulous attention to presentation, with modifications such as intraorbital inserts, which were probably intended to enhance lifelike appearance. These observations situate Gorini’s work within a broader 19th-century scientific and cultural context, in which anatomical experimentation intersected with public display, commemoration, and the pursuit of permanence over mortality.

Beyond the technical aspects, the study underscores the cultural and emotional significance of child mummification. The decision to preserve non-adult individuals possibly reflects contemporary attitudes toward childhood, death, and memory in a period marked by high infant mortality rates. The Gorini collection thus provides not only a window into scientific practices of the time but also a unique testimony to how 19th-century society negotiated the tension between grief and the desire for preservation and memorialization.

Finally, the research presented here also contributes to the future safeguarding of the Gorini collection. By documenting the current condition of the specimens and assessing previous conservation treatments, this study lays the groundwork for informed preservation strategies. Continued interdisciplinary approaches—combining radiology, anthropology, chemistry, and conservation science—will be essential to ensure the long-term stability of these unique remains, while also deepening our understanding of Gorini’s legacy and the complex interplay between science, culture, and mortality in the late modern era.

## Data Availability

The original contributions presented in the study are included in the article/supplementary material, further inquiries can be directed to the corresponding author.
